# Mannosyl-Recognizing Receptors Induce an M1-Like Phenotype in Macrophages of Susceptible Mice but an M2-Like Phenotype in Mice Resistant to a Fungal Infection

**DOI:** 10.1371/journal.pone.0054845

**Published:** 2013-01-30

**Authors:** Claudia Feriotti, Flávio V. Loures, Eliseu Frank de Araújo, Tania Alves da Costa, Vera L. G. Calich

**Affiliations:** Departamento de Imunologia, Instituto de Ciências Biomédicas, Universidade de São Paulo, São Paulo, São Paulo, Brazil; Institut National de la Santé et de la Recherche Médicale, France

## Abstract

In addition to alpha1,3 glucan, mannan and mannan-linked proteins are expressed in the outer layer of *Paracoccidioides brasiliensis* yeasts. The recognition of mannosyl residues by multiple pathogen recognition receptors, such as the mannose receptor (MR), complement receptor 3 (CR3) and toll-like receptor 4 (TLR4) on macrophage membranes can influence macrophage activation and the mechanisms of innate immunity against fungal pathogens. The aim of this study was to clarify the role of these receptors in the interaction between *P. brasiliensis* and macrophages from resistant (A/J) and susceptible (B10.A) mice. Therefore, the phagocytic, fungicidal and secretory abilities of macrophages were evaluated in the presence of mannan and antibodies against MR, CR3 and TLR4. We verified that mannan increased and anti-MR antibody decreased the killing ability and nitric oxide production of macrophages. The specific blockade of MR, CR3 and TLR4 by monoclonal antibodies impaired fungal recognition and modulated the production of cytokines. Mannan or *P. brasiliensis* induced decreased expression of MR and TLR2 on A/J macrophages, whereas CR3, TLR4 and TLR2 were reduced on B10.A cells. Importantly, both mannan and *P. brasiliensis* induced the production of IL-12 by B10.A macrophages, whereas TGF-β, TNF-α and IL-6 were produced by A/J cells. In addition, B10.A macrophages exhibited a prevalent expression of inducible NO-synthase and SOCS3 (suppressor of cytokine signaling-3), indicating a pro-inflammatory, “M1-like” differentiation. In contrast, the elevated expression of arginase-1, found in inflammatory zone-1 (FIZZ1), YM1 (CHI313, chitinase-like lectin), and SOCS1, typical markers of alternatively activated macrophages, indicates a prevalent “M2-like” differentiation of A/J macrophages. In conclusion, our data reveal that several mannosyl-recognizing receptors coordinate the apparently paradoxical innate response to paracoccidioidomycosis, in which resistance is initially mediated by alternatively activated phagocytes and tolerance to fungal growth, whereas susceptibility is linked to classically activated macrophages and the efficient control of fungal growth.

## Introduction

Toll-like receptors (TLRs) and the C-type lectin receptors (CLRs) are important pathogen recognition receptors (PRRs) expressed by the cells of the innate immune system. Their primary function is to sense the invasion of microorganisms [1]. The interactions between the pathogen associated molecular patterns (PAMPs) of microorganisms and the PRRs of phagocytic cells play a fundamental role in the type and efficiency of innate and adaptive immune mechanisms that develop in response to these interactions [2]–[9]. The mannose receptor (MR), a member of the C-type lectin family, is a multifunctional endocytic receptor present in most tissue macrophages (MØs) and in hepatic and lymphatic endothelia. Importantly, the MR is also expressed by subsets of dendritic cells that mediate antigen uptake, leading to enhanced presentation to T cells [2], [5], [10], [11]. In addition to the MRs, other membrane PRRs, such as TLR4, TLR2, CR3 (CD11b/CD18), dendritic cell-specific intercellular adhesion molecule-3-grabbing non-integrin (DC-SIGN, CD209) and dectin-2, interact with mannosyl residues [12]–[15]. Furthermore, a recent study demonstrated that TLR4 recognizes O-linked mannosyl residues, whereas MR interacts with N-linked residues on the cell wall of *Candida albicans* [16]. Another mannan-containing compound, the phospholipomannan of *C. albicans*, is a cell wall ligand for TLR2 [17].

*Paracoccidioides brasiliensis*, the causative agent of human paracoccidioidomycosis, is primarily a respiratory pathogen, and the host is infected through inhalation of airborne spores. A great majority of infected subjects develop an asymptomatic pulmonary infection, although some individuals present clinical manifestations that give rise to the adult or the juvenile forms of the disease. The adult form of PCM is the most common form; it has a chronic evolution, and mild or severe cases are associated with Th1- or Th2-biased patterns of immunity, respectively. The juvenile or acute form of PCM is usually severe, induces a profound anergy of DTH reactions and is associated with a mixed Th2/Th3 pattern of T cell activation [18], [19].

Our laboratory has established an experimental murine model of pulmonary PCM. In this model, A/J and B10.A mice are resistant and susceptible to *P. brasiliensis* infection, respectively. The A/J mice develop chronic, benign pulmonary-restricted paracoccidioidomycosis, coupled with well-organized lesions containing a low number of yeasts and positive DTH reactions resembling those in the benign form of PCM. In contrast, the B10.A mice develop a progressive disseminated disease associated with increasing fungal loads, DTH anergy, and non-organized lesions mimicking those in the severe forms of PCM [20], [21]. The susceptibility of B10.A mice was associated with CD4^+^ T cell anergy and prevalent CD8^+^ T cell response. In resistant mice, protective T cell immunity develops late in infection and was shown to be mediated by CD4^+^ and CD8^+^ T lymphocytes secreting a mixed pattern of type 1 and 2 cytokines. Interestingly, during the innate phase of immunity, alveolar macrophages from susceptible mice exhibit better control of *P. brasiliensis* growth than those from resistant hosts. However, during later phases, only A/J macrophages are able to control fungal loads, and this behavior paralleled the development of robust DTH reactions and IFN-γ secretion [22], [23]. The early enhanced fungicidal ability of the B10.A macrophages was associated with elevated IL-12 and nitric oxide (NO) production, but NO was also involved in the early T cell anergy developed by this mouse strain. Alveolar macrophages from A/J mice secreted low levels of NO concomitantly with high levels of TGF-β, resulting in poor fungicidal activity [23], [24]. These divergent patterns of macrophage activation profoundly influence the adaptive immunity subsequently developed by B10.A and A/J mice, leading us to hypothesize that different PRRs may be implicated in their responses to *P. brasiliensis* infection.

TLRs, CLRs and components of the integrin family of receptors, such as CR3 (CD11b/CD18), a complement receptor that binds to iC3b, are important in the resistance to fungal infections [16], [25]. We were the first to demonstrate that the interaction of *P. brasiliensis* with peritoneal macrophages was enhanced by iC3b opsonization of yeast cells [26]. Our recent work has also demonstrated that TLR2, TLR4 and MyD88 signaling are involved in the initial interaction between alveolar and peritoneal macrophages with yeast cells [27]–[29]. CR3 and MR recognize *P. brasiliensis* conidia [30], while gp43, the immunodominant antigen of *P. brasiliensis*, was shown to bind to the MRs and inhibit the phagocytic and fungicidal ability of peritoneal macrophages from susceptible and resistant mice [31]. Two recent studies with human monocytes have also suggested that MR, TLR2, TLR4, and dectin-1 contribute to the recognition of gp43 and *P. brasiliensis* yeasts, indicating important roles for these pathogen receptors in the immune response against the fungus [32], [33].

Because mannan-rich components are present in the outer layer of the *P. brasiliensis* cell wall [34]–[37], and because MRs seem to be involved in the recognition of gp43 and fungal spores, we decided to further and concomitantly explore the role of diverse mannosyl recognizing receptors in the interaction of *P. brasiliensis* yeasts with macrophages from resistant and susceptible mice. In A/J macrophages, mannan treatment or *P. brasiliensis* infection led to decreased expression of MR and TLR2, increased production of TNF-α, IL-6 and TGF-β. These treatments led also to increased expression of several signature genes of alternatively activated macrophages [(arginase-1, found in inflammatory zone 1 (FIZZ1), YM1 (CHI313), chitinase-like lectin, and silencing of cytokines synthesis-1 (SOCS1)], demonstrating a prevalent M2-like pattern of differentiation. In contrast, the same treatments in B10.A cells resulted in reduced expression of CR3, TLR4 and TLR2, increased production of IL-12 and increased expression of induced NO-synthase (NOS2) and SOCS3, indicating a prevalent pro-inflammatory activation, or M1-like differentiation. These opposite patterns of *P. brasiliensis* recognition and macrophage activation driven by mannosyl-recognizing receptors appear to play a pivotal role in the divergent innate and adaptive immunities developed by resistant and susceptible mice in response to this primary fungal pathogen. The initial tolerogenic response of resistant mice evolves to efficient adaptive immunity, highly controlled by strong Treg cells which appear to avoid excessive inflammation and tissue pathology, whereas the efficient innate immunity of B10.A mice promoted by excessive NO production leads to suppressed T cell immunity, progressive disseminated disease and fatal outcome [38].

## Materials and Methods

### Ethics Statement

Animal experiments were performed in strict accordance with the Brazilian Federal Law 11,794 establishing procedures for the scientific use of animals, and the State Law establishing the Animal Protection Code of the State of São Paulo. All efforts were made to minimize suffering, and all animal procedures were approved by the Ethics Committee on Animal Experiments of the Institute of Biomedical Sciences of University of São Paulo (Proc.76/04/CEEA).

### Fungus

Pb18 *P. brasiliensis*, originally isolated from a young patient in 1929, was a gift from Prof. C. Fava Netto. The isolate was maintained by weekly subcultivation in semisolid Fava Netto’s medium at 35°C [39]. Virulence was maintained by in vivo passages as described by Kashino et al. [40]. Yeast cells were harvested, washed and adjusted to 8×10^4^ cells/mL based on hemocytometer counts. Viability was determined with Janus Green B vital dye (Merck Frankfurter Straße, Darmstadt, GER) and was always higher than 85%. All solutions used to prepare yeast cell suspensions and macrophages were tested for the presence of LPS using the *Limulus* amoebocyte lysate chromogenic assay (Sigma-Aldrich St. Louis, MO, USA) and always showed LPS levels <0.015 EU/mL.

### Mice

Susceptible (B10.A) and resistant (A/J) mouse strains to *P. brasiliensis* infection were obtained from our Isogenic Unit (Immunology Department of Institute of Biomedical Sciences of University of São Paulo, Brazil) and used at 8 to 11 weeks of age. SPF mice were fed sterilized laboratory chow and water ad libitum.

### PI Labeling of *P. brasiliensis* Yeast Cells

*P. brasiliensis* yeast cells were washed in PBS and heat killed at 60°C for 1 h. Before the labeling, the yeast suspension was sonicated using 3 cycles of 10 s each (21% amplitude) with Sonics (Vibra Cell VCX 750; Sonics & Materials, Newtown, CT, USA) to eliminate aggregates. The yeast cells were washed, adjusted to 1×10^6^ cells/mL in PBS, and then incubated with propidium iodide (PI; 100 µg/ml; Sigma) for 30 min at 37°C. The yeast suspension was then washed three times with PBS and stored at 4°C.

### Phagocytic and Fungicidal Assays

To induce peritoneal macrophages, B10.A and A/J mice were injected with 3 mL of Brewer thioglycollate medium (Difco laboratories, Detroit, MI, USA). Three days later, the mice were injected with 5 mL of cold PBS, the peritoneal content was extracted, and the cells were washed. Macrophages were isolated by adherence (2 h at 37°C in 5% CO_2_) to plastic-bottom tissue-culture plates (1×10^6^ cells/well in 24 well plates for fungicidal assays), or plated onto 13-mm-round glass coverlips (1×10^6^ cells/well in 24 well plates for phagocytosis). Macrophages were washed to remove nonadherent cells and cultivated overnight with fresh complete medium (DMEM, Dulbecco’s Modified Eagle’s Medium, Sigma), containing 10% heat inactivated fetal calf serum, 100 U/ml penicillin and 100 µg/ml streptomycin) in the presence or absence of recombinant IFN-γ (20 ng/ml in culture medium, BD-Pharmingen San Diego, CA, USA). Non-adherent cells were counted to evaluate the number of remaining adherent cells used in phagocytic and killing assays. Macrophage cultures were incubated with mannan from *Saccharomyces cereviseae* (Sigma, 2.5, 0.5 and 0.1 mg/ml) for 30 min. at 37°C in 5% CO_2_, and then infected with *P. brasiliensis* yeasts in a macrophage:yeast ratio of 25∶1. This ratio was previously determined and was shown to be non deleterious to macrophage cultures and adequate for phagocytosis and killing assays [41], [42]. In selected experiments, instead of mannan treatment, macrophages monolayers were incubated with anti-MR (Serotec Raleigh, NC, USA, 20 µg/ml), anti-CD11b (e-Bioscience San Diego, CA, USA, 10 µg/ml) or anti-TLR4 antibodies (e-Bioscience, 10 µg/ml) by 30 min. at 37°C, and then infected with yeast cells. For phagocytic assays, the cells were co-cultivated for 4 h at 37°C in 5% CO_2_ to allow fungi adhesion and ingestion. Cells were gently washed twice with PBS to remove any non-ingested or non-adhered yeasts, and samples were processed for microscopy. Cells were fixed with methanol and stained with Giemsa (Sigma). Experimental conditions were performed in triplicate, and the number of phagocytosed or adhered yeasts per 1,000 macrophages was evaluated on at least three separate slides. For some phagocytic assays, mannan- treated (2,5 mg/mL) and untreated macrophages from A/J and B10.A mice were infected with heat-inactivated, propidium iodide (PI) labeled *P. brasiliensis* yeasts at a macrophage:yeast ratio of 1∶1 for 2 h as previously described [29]. Macrophages were detached from plastic with fresh cold medium and a rubber cell scraper on ice. The cells were transferred to tubes, centrifuged (400×g. 10 min., 4°C), and the pellets were labeled with 2 µL of anti-F4/80 (APC) antibodies diluted in PBS in a final volume of 25 µL per tube and incubated for 20 min. at 4°C. The cells were washed twice in PBS, the pellets were suspended in 200 µL of PBS 1% FCS and were immediately read on FACScalibur (Becton Dickinson, Franklin Lakes, NJ, USA) and data analyzed using the FlowJo software program (Tree Star, Inc., Ashland, OR, USA). For fungicidal assays, IFN-γ-primed and unprimed macrophage cultures were treated with mannan and infected with *P.brasiliensis* yeasts as above described. After 48 h of culture at 37°C in a CO_2_ incubator, plates were centrifuged (400×*g*, 10 min, 4°C), supernatants stored at –70°C and further analyzed for the presence of nitrite and cytokines. The wells were washed with distilled water to lyse macrophages, and suspensions collected in individual tubes. One hundred µl of cell homogenates were assayed for the presence of viable yeasts. All assays were done with five wells per condition in over three independent experiments.

### Assay for CFU

Coculture homogenates (100 µl) were plated on brain heart infusion agar (Difco, San Diego, CA, USA), which contained 4% (vol/vol) normal horse serum (Instituto Butantan, São Paulo, SP) and 5% *P. brasiliensis* 192 culture filtrate, the latter constituting the source of growth-promoting factor [43]. When necessary, dilutions were made in sterile PBS. The plates were incubated at 35°C, and colonies were counted daily until no increase in counts was observed. The numbers of viable *P. brasiliensis* are expressed as the means ±SE.

### Measurements of Cytokines and NO

Supernatants from cell cultures were separated and stored at −70°C. The levels of IL-6, IL-12, IL-10, tumor necrosis factor alpha (TNF-α) and tissue growth factor-beta (TGF-β) were measured by a capture enzyme-linked immunosorbent assay (ELISA) with antibody pairs purchased from BD Pharmingen (San Diego, CA, USA). The ELISA procedure was performed according to the manufacture’s protocol, and absorbance was measured with a Versa Max Microplate Reader (Molecular Devices, Sunnyvale, CA, USA). The concentrations of cytokines were determined with reference to a standard curve for serial twofold dilutions of murine recombinant cytokines. Nitric oxide production was quantified by the accumulation of nitrite in the supernatants from *in vitro* protocols by a standard Griess reaction. All determinations were performed in duplicate, and results were expressed as micro molar concentration of NO.

### Flow Cytofluorometry

IFN-γ-primed (20 ng/ml) or unprimed B10.A and A/J macrophages were treated or untreated with mannan from *Saccharomyces cereviseae* (2.5 mg/ml) for 30 min. at 37°C in 5% CO_2_. Cells were then infected with *P. brasiliensis* yeasts in a macrophage:yeast ratio of 25∶1 for 2 h in 24-well culture plates. Cultures were gently washed and cultivated for an additional 12 h period. Supernatants were removed, cultures were washed, and macrophages were detached from plastic plates with fresh cold medium and a rubber cell scraper. Cells were adjusted to 5×10^5^ viable cells/ml in staining buffer (PBS with 0.1% NaN_3_ and 1% fetal calf serum). Fc receptors were blocked by the addition of unlabeled anti-CD16/32 (Fc block; BD Pharmingen).After Fc receptor blocking, cells were stained for 20 min at 4°C. APC-labeled monoclonal antibodies against F4/80; Phycoerythrin (PE)-labeled monoclonal antibodies against CD11b, TLR2 and Dectin-1 and ALEXA 488-anti-MR and TLR4 monoclonal antibodies (BD Bioscience) were used. Cells were fixed with 1% paraformaldehyde (Sigma) and were stored in the dark at 4°C until analysis in a flow cytometer. The acquisition and analysis gates were restricted to the macrophages. A minimum of 100,000 events were acquired and the expression of macrophage markers on the cell surface was analyzed in a FACS Canto flow cytometer (BD Pharmingen) using the FlowJo software program (Tree Star, Inc., Ashland, OR, USA).

### Quantitative Real-Time PCR

Total RNA was extracted from cultures of normal or *P. brasiliensis*-infected macrophages before and after mannan treatment using the TRIzol reagent (Invitrogen, Life Technologies, Carlsbad, CA, USA) according to the manufacturer’s instructions. The RNA concentrations were determined by spectrophotometer readings at an absorbance of 260 nm. First-strand cDNAs were synthesized from 2 µg RNA using the High Capacity RNA-to-cDNA kit (Applied Biosystems, Foster City, CA, USA) according to the manufacturer’s instructions. Real-time polymerase chain reaction (RT-PCR) was performed using the TaqMan real-time PCR assay (Applied Biosystems, Life Technologies) for the following molecules: SOCS3 (Mm00545913_s1), SOCS1 (Mm00782550_s1), ARG1 (Mm00475988_m1), NOS2 (Mm00440502_m1), FIZZ or Retnla (Mm00445109_m1), and Ym1 or Chi3l3 (Mm 00657889_mH). Cycling conditions were as follows: 10 min at 95°C, followed by 45 cycles of 20 s at 95°C, 20 s at 58°C, and 20 s at 72°C. Analysis was performed with the ABI PRISM 7000 sequence detection system (Applied Biosystems). GAPDH was used as an internal control. All values were normalized to GAPDH, and the relative gene expression was calculated using the Pfaffl method [44].

### Statistical Analysis

Data were expressed as the mean ± SEM. Differences between groups were analyzed by Student’s *t* or analysis of variance (ANOVA) followed by the Tukey test using PRISMA 5.04 software (GraphPad, SanDiego, CA, USA). *P* values under 0.05 were considered significant.

## Results

### Mannan Treatment Decreases the Phagocytic Ability of Macrophages

**Figure 1:**
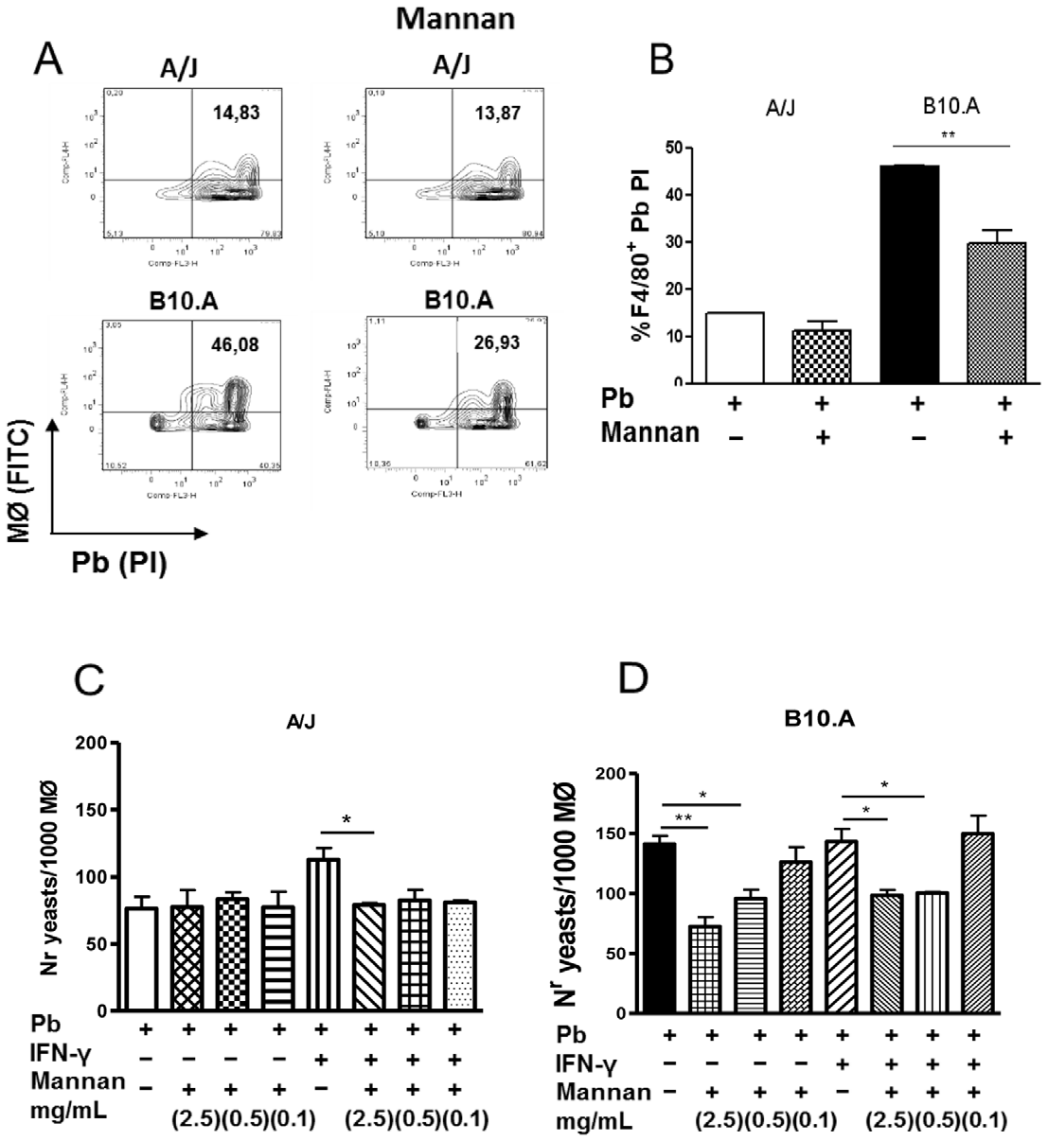
Mannan treatment induces a decreased phagocytic ability of B10.A and A/J macrophages. (A, B) Macrophages from A/J and B10.A mice were previously treated by mannan (2.5 mg/ml). Cells were then infected with *P. brasiliensis* yeasts labeled with propidium iodide (1∶1, fungus:macrophage ratio) for 2 h. Co-cultures were gently washed and macrophages labeled with anti-F4/80 (APC) antibodies for 20 min., and immediately analyzed on a FACSCalibur. (C, D) Phagocytosis assays were also performed in round glass coverlips; macrophages cultures (1×10^6^/well) were primed or unprimed with IFN-γ (20 ng/ml), treated or untreated with mannan (2.5, 0.5 and 0.1 mg/mL) and then infected with 4×10^4^ viable yeasts (1∶25 fungus: macrophages ratio). After 4 h incubation supernatants were aspirated, the monolayer gently washed with warm PBS and the cells stained with Giemsa. An average of 1,000 macrophages was counted and the number of ingested and/or adherent yeasts was determined. Data are means ± SEM of triplicate samples from two experiment determinations. (*P<0.05 and **P<0.01).

Prior to performing fungicidal studies, we asked whether mannan treatment would interfere with the phagocytic ability of macrophages from resistant and susceptible mice. A phagocytic activity assay was carried out using propidium iodide (PI)-labeled *P. brasiliensis.* Macrophages previously treated with mannan (2.5 mg/mL) or untreated were cultivated at a macrophage:yeast ratio of 1∶1 for 2 h at 37°C. Cultures were washed and macrophages detached from plastic plates using fresh, cold medium and a rubber cell scraper. Macrophages were labeled with an anti-F4/80 (APC) monoclonal antibody for 20 min at 4°C and the samples were read on a FACS Calibur flow cytometer. As shown in Figure 1A and B, mannan treatment diminished the phagocytic activity of B10.A macrophages (46.08% vs. 26.93%, P<0.01). However, this treatment did not induce a significant change in the numbers of fungal cells associated (ingested/adhered) with A/J macrophages (14.83% vs. 13.87%). The phagocytic assay was also performed on round glass coverslips using IFN-γ primed and unprimed macrophages. Macrophage cultures (1×10^6^/well) were pre-activated by IFN-γ (20 ng/mL) or not and then treated with three different mannan concentrations. These cells were then infected with 4×10^4^ viable yeasts (1∶25 fungus:macrophage ratio). After a 4 h incubation, supernatants were aspirated, the monolayer gently washed with PBS and the cells stained with Giemsa. An average of 1,000 macrophages was counted, and the number of ingested and/or adherent yeasts was determined (Figure 1C, D). As shown in Figure 1C, only the highest mannan concentration (2.5 mg/mL) decreased the phagocytic activity of IFN-γ activated A/J macrophages. In contrast, mannan solutions at concentrations of 2.5 and 0.5 mg/ml were able to inhibit the phagocytic activity of both IFN-γ -primed and unprimed B10.A macrophages (Figure 1D). Therefore, in both phagocytic assays, the B10.A macrophages showed higher phagocytic/adherence ability than the A/J cells, and this ability was markedly influenced by mannan treatment.

### Mannan Treatment Increases the Fungicidal Activity and NO Production of IFN-γ-primed B10.A and A/J Macrophages

**Figure 2:**
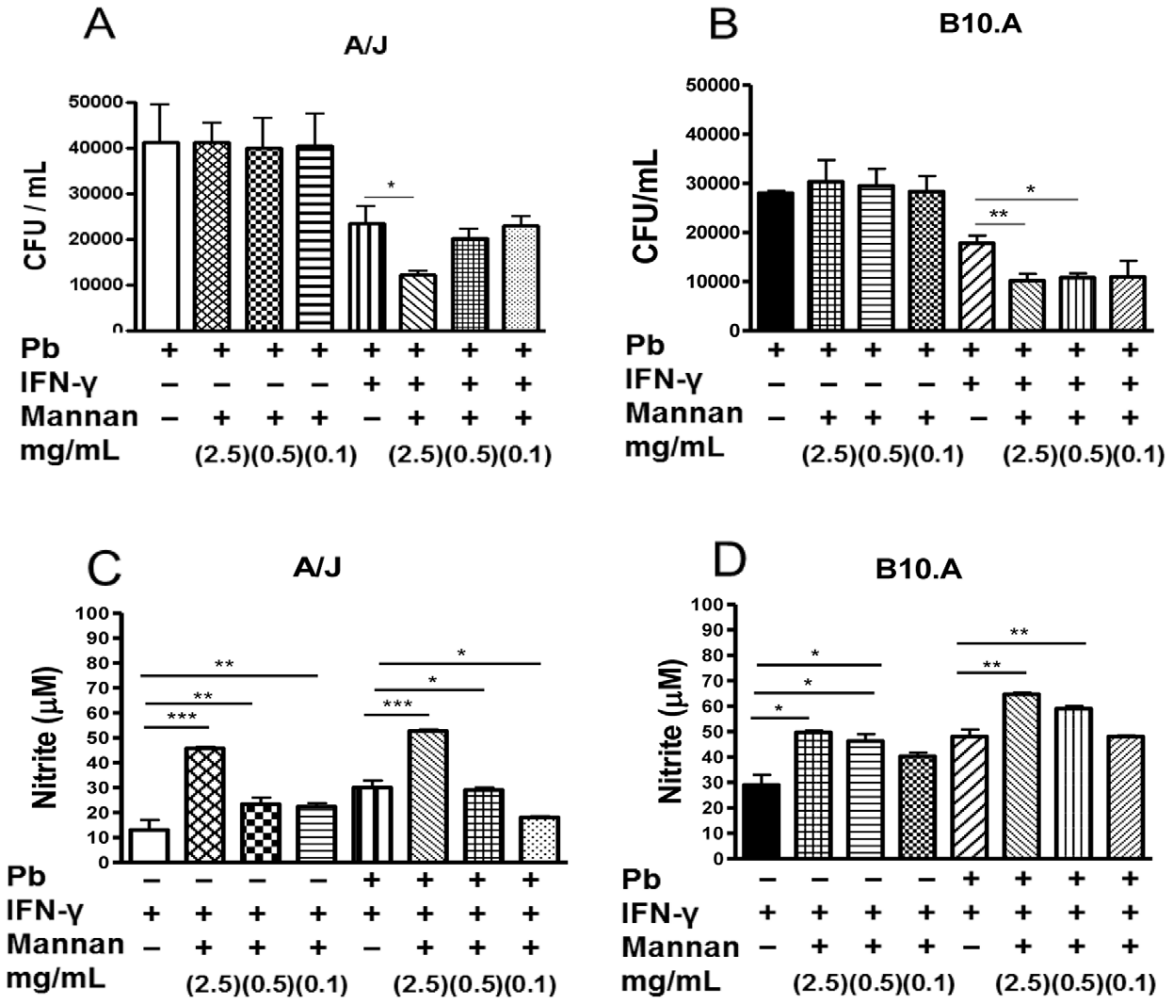
Mannan treatment induced increased fungicidal ability and NO production by macrophages from resistant (A/J) and susceptible (B10.A) mice. (A, B) CFU assays were performed to determine the recovery of viable fungi in cell homogenates. Macrophages were primed or unprimed with IFN-γ (20 ng/mL) overnight, treated by mannan (2.5, 0.5 and 0.1 mg/mL) and then challenged with viable *P. brasiliensis* yeasts (1∶25, fungus:macrophages ratio). Two hours later the cultures were gently washed, cultivated for an additional 48 h period, and the number of recovered viable yeasts measured by a CFU assay. (C, D) Nitric oxide (NO) production was measured in culture supernatants by a Griess reagent. Data are means ± SEM of triplicate samples from two experiment determinations. (*P<0.05, **P<0.01 and ***P<0.001).

For the fungicidal assay, untreated and mannan-treated macrophages (2.5, 0.5 and 0.1 mg/mL) were cultivated with *P. brasiliensis* yeasts for 48 h. Supernatants were removed and assayed for the presence of nitric oxide and cytokines, and the cell homogenates were plated for CFU determinations. In both mouse strains, mannan treatment did not induce a significant change in the recovery of viable yeasts from IFN-γ -unprimed macrophages (Figure 2A, B). In contrast, fewer viable yeasts were obtained from IFN-γ -primed B10.A and A/J macrophages (Figure 2A, B). This effect was more prominent with B10.A macrophages, which showed increased fungicidal activity at two different mannan concentrations (2.5 and 0.5 mg/mL, P<0.01 and P<0.05). In addition, *P. brasiliensis* infection and mannan treatment led to an increase in NO production by IFN-γ -primed macrophages (Figure 2 C, D). A dose-response effect was observed at the three different mannan concentrations employed; however, a more prominent NO production was detected in the B10.A macrophages. Prior IFN-γ priming was a prerequisite for NO production because unprimed macrophages of both mouse strains did not secrete IFN-γ (data not shown). These results suggested that mannan can activate the fungicidal activity and NO production of both the B10.A and the A/J macrophages. However, both responses were only detected when the macrophages were primed with IFN-γ.

### Mannan and *P. brasiliensis* Induce Similar Patterns of Cytokines Secreted by A/J and B10.A Macrophages

**Figure 3:**
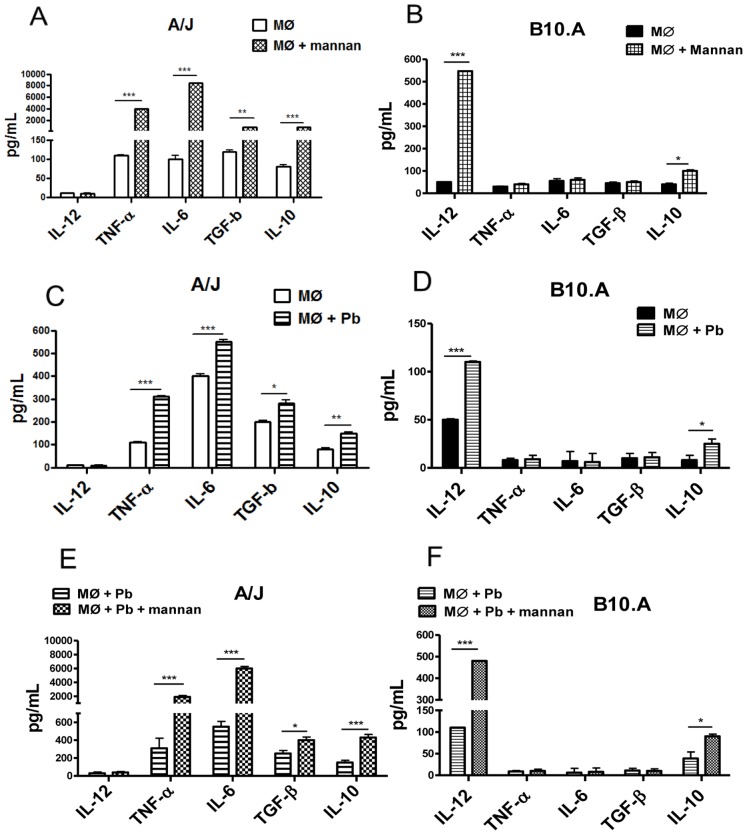
Mannan treatment leads to increased IL-12 production by B10.A macrophages but induces increased levels of IL-6, TNF-α, and TGF-β production by A/J macrophages. (A, B) Macrophages were untreated or treated by mannan (2.5 mg/mL) for 30 min and cultivated for 48 h. (C, D) Some cultures were only infected by viable *P. brasiliensis* yeasts (1∶25, fungus:macrophages ratio) for 48 h. (E, F) Macrophages were treated by mannan (30 min) and then infected (48 h) with yeast cells. Supernatants were removed and used for cytokines measurements by ELISA. Data are means ± SEM of triplicate samples from two experiment determinations. (*P<0.05, **P<0.01 and ***P<0.001).

To further characterize the effect of mannan on the interaction of *P. brasiliensis* with macrophages from resistant and susceptible mice, supernatants from the killing assays were tested for the presence of some macrophage-activating cytokines (IL-12, IL-6 and TNF-α) and the macrophage-deactivating cytokines (IL-10 and TGF- β). As depicted in Figure 3A, mannan-treated macrophages from the A/J mice secreted higher levels of IL-6, TNF-α and TGF-β, whereas the B10.A macrophages secreted higher levels of IL-12, compared to untreated macrophages (Figure 3B). Increased concentrations of IL-10 were detected in both the A/J and B10.A macrophages. Similar results were found with IFN-γ-primed macrophages (data not shown). IL-23 and IL-1β were also measured in macrophage supernatants; however, no significant production of these cytokines was detected. *P. brasiliensis* infection induced a similar pattern of cytokine secretion as mannan treatment, albeit at a lower level (Figure 3C, D). Moreover, the type and levels of cytokines produced by mannan-treated cultures infected with *P. brasiliensis* were similar to those induced by mannan treatment alone (Figure 3E, F).

### A/J and B10.A Macrophages Express Different Levels of Costimulatory Molecules and Phagocytic and Non-phagocytic PRRs

**Figure 4:**
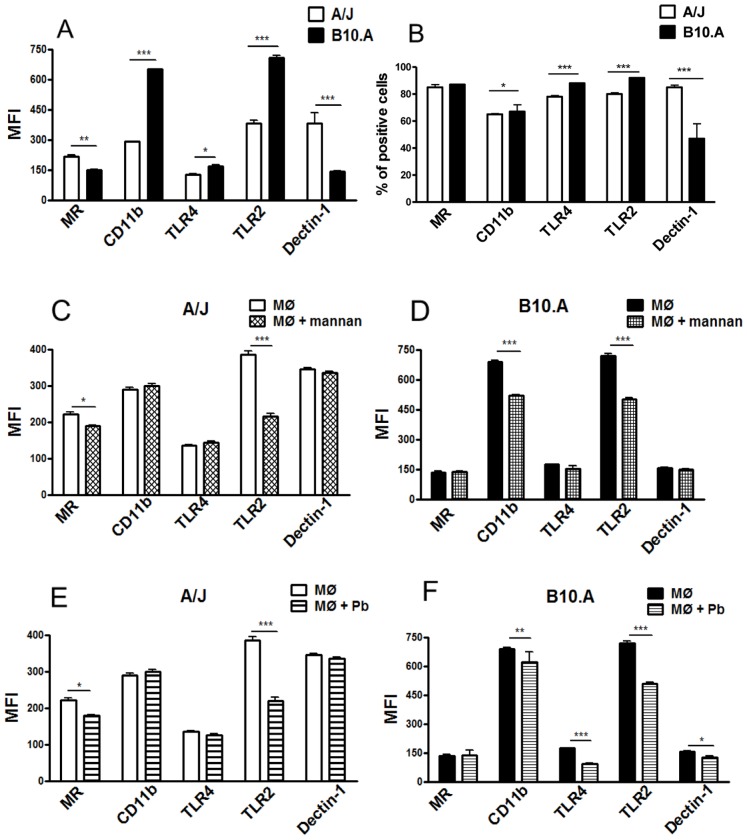
Macrophages from resistant (A/J) and susceptible (B10.A) mice express different levels of pathogen recognizing receptors (PRRs) which are differently mobilized upon *P. brasiliensis* infection. The Median Fluorescence Intensity (MFI) and frequency of positive macrophages expressing MR, CD11b (CR3), TLR4, TLR2 and dectin-1 were measured by flow cytometry. The macrophage suspensions were obtained and stained as described in materials and methods. The acquisition and analysis gates were restricted to F4/80 labeled macrophages population. (A, B) MFI and percentage of PRRs expressed by normal A/J and B10.A macrophages. (C, D) PRRs expression by mannan treated (2.5 mg/mL for 30 min) macrophages. (E, F) PRRs expression by *P. brasiliensis* infected macrophages (1∶25, fungus:macrophages ratio). Data are means ± SEM of triplicate samples from two experiment determinations. (*P<0.05, **P<0.01 and ***P<0.001).

The different behavior of A/J and B10.A macrophages upon *P. brasiliensis* infection and mannan treatment led us to hypothesize that different receptors could be involved in this interaction. Indeed, TLR4, TLR2 and CR3 molecules, in addition to MRs, were reported to interact with the mannosyl residues of mannose-containing molecules [9], [17], [25]. Therefore, we assessed the expression of these and other surface molecules on normal, unstimulated B10.A and A/J macrophages. The expression levels of the phagocytic receptors MR and dectin-1, and the non-phagocytic receptors TLR4, TLR2, and CR3 (CD11b/CD18) were assessed by flow cytometry on normal, untreated B10.A and A/J macrophages (Figure 4A, B). Compared to the A/J macrophages, the B10.A cells showed increased levels of CD11b, TLR4 and TLR2, as determined by MFI, and increased percentages of cells expressing these receptors. In contrast, A/J macrophages showed increased expression of MR and dectin-1 (Figure 4A, B). Similar results were obtained with IFN-γ-primed macrophages (data not shown).

### Both, *P. brasiliensis* Infection and Mannan Treatment Reduce the Expression of MR and TLR2 on A/J Macrophages and Reduce the Expression of CR3, TLR4 and TLR2 on B10.A Macrophages

We used flow cytometry to further characterize the differences in the expression of surface molecules on B10.A and A/J macrophages upon *P. brasiliensis* infection and mannan treatment. Normal and IFN-γ-primed macrophages from A/J and B10.A mice previously treated with mannan (2.5 mg/mL) and/or infected with *P. brasiliensis* were cocultivated for 12 h. The expression levels of MR, TLR4, TLR2, dectin-1 and CR3 (CD11b) were then assayed by flow cytometry. The acquisition and analysis gates were restricted to the F4/80 labeled macrophage population. As seen in Figure 4C and D, mannan treatment decreased the expression of MR and TLR2 on A/J macrophages, whereas CR3 and TLR2 expression levels were lower on B10.A macrophages. Importantly, *P. brasiliensis* infection resulted in an equivalent reduction of MR and TLR2 on A/J macrophages and CR3, TLR4 and TLR2 on B10.A cells (Figure 4E, F). Similar results were found with IFN-γ-primed macrophages (data not shown). Once again, mannan treatment and *P. brasiliensis* infection resulted in similar changes in the expression of receptors on macrophages, suggesting an important role for mannosyl-recognizing receptors in the initial interaction of macrophages with *P. brasiliensis* yeasts.

### MR Blockade by Anti-MR Antibodies Decreases the Phagocytic Activity, Killing Activity and NO Production of A/J and B10.A Macrophages

**Figure 5:**
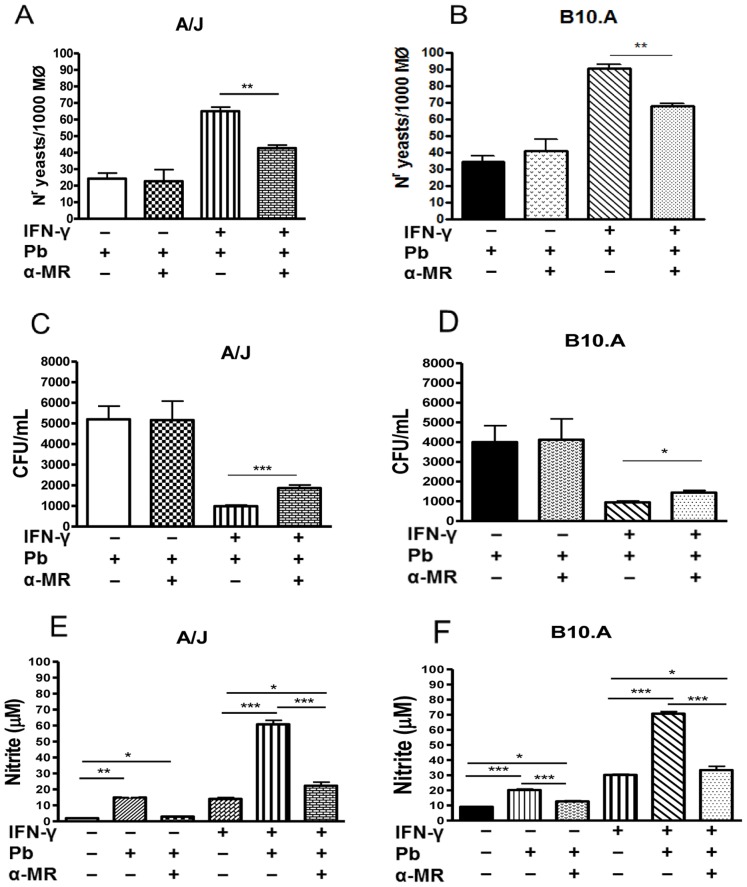
MR blockade by anti-MR antibodies decreases the phagocytic and fungicidal abilities and NO production of A/J and B10.A macrophages. (A, B) Phagocytic assays were performed in round glass coverlips as described before. Macrophages cultures (1×10^6^/well) were primed or unprimed with IFN-γ (20 ng/ml) overnight followed by blockade with anti-MR antibody (20 µg/mL) for 30 min. Cells were then infected with *P. brasiliensis* yeasts (1∶25 fungus: macrophage ratio) for 4 h. An average of 1,000 macrophages was counted and the number of ingested and/or adherent yeasts was determined in Giemsa stained coverslips. (C, D) Some macrophages cultures were treated by anti-MR antibodies, infected with *P. brasiliensis* (1∶25, fungus:macrophages ratio) and cultivated for 48 h. The fungicidal activity was determined by a CFU assay using cell homogenates. (E, F) NO production was measured in culture supernatants by the Griess reaction. Data are means ± SEM of triplicate samples from two experiment determinations. (*P<0.05, **P<0.01 and ***P<0.001).

To further characterize the involvement of MRs in the interaction of *P. brasiliensis* with B10.A and A/J macrophages, this receptor was blocked by anti-MR specific antibodies (20 µg/ml) and the phagocytic and fungicidal abilities of the cells were then analyzed. As shown in Figure 5A and B, anti-MR antibody treatment decreased the number of yeasts associated (ingested/adhered) with IFN-γ-primed macrophages from both the A/J and B10.A mice. However, the same treatment did not alter the behavior of IFN-γ untreated macrophages. For the fungicidal assay, macrophages were cultivated with *P. brasiliensis* yeasts for 48 h. Supernatants were then removed and assayed for the presence of nitric oxide and cytokines, and the cell homogenates were plated for CFU determinations. The blockade of MRs by anti-MR antibodies increased the recovery of viable yeasts from IFN-γ-primed but not unprimed macrophages from both mouse strains (Figure 5C, D). In addition, MR blockade led to diminished levels of NO in both mouse strains and in both primed and unprimed macrophages, (Figure 5E, F). Together, these data demonstrate that the MR plays an important role in the interaction of *P. brasiliensis* yeasts with B10.A and A/J macrophages by regulating their IFN-γ-induced phagocytic and fungicidal abilities and also by controlling NO production.

### MR Blockade by Anti-MR Antibodies Abrogates TNF-α and IL-6 Production by A/J Macrophages and IL-12 Production by B10.A Cells

**Figure 6:**
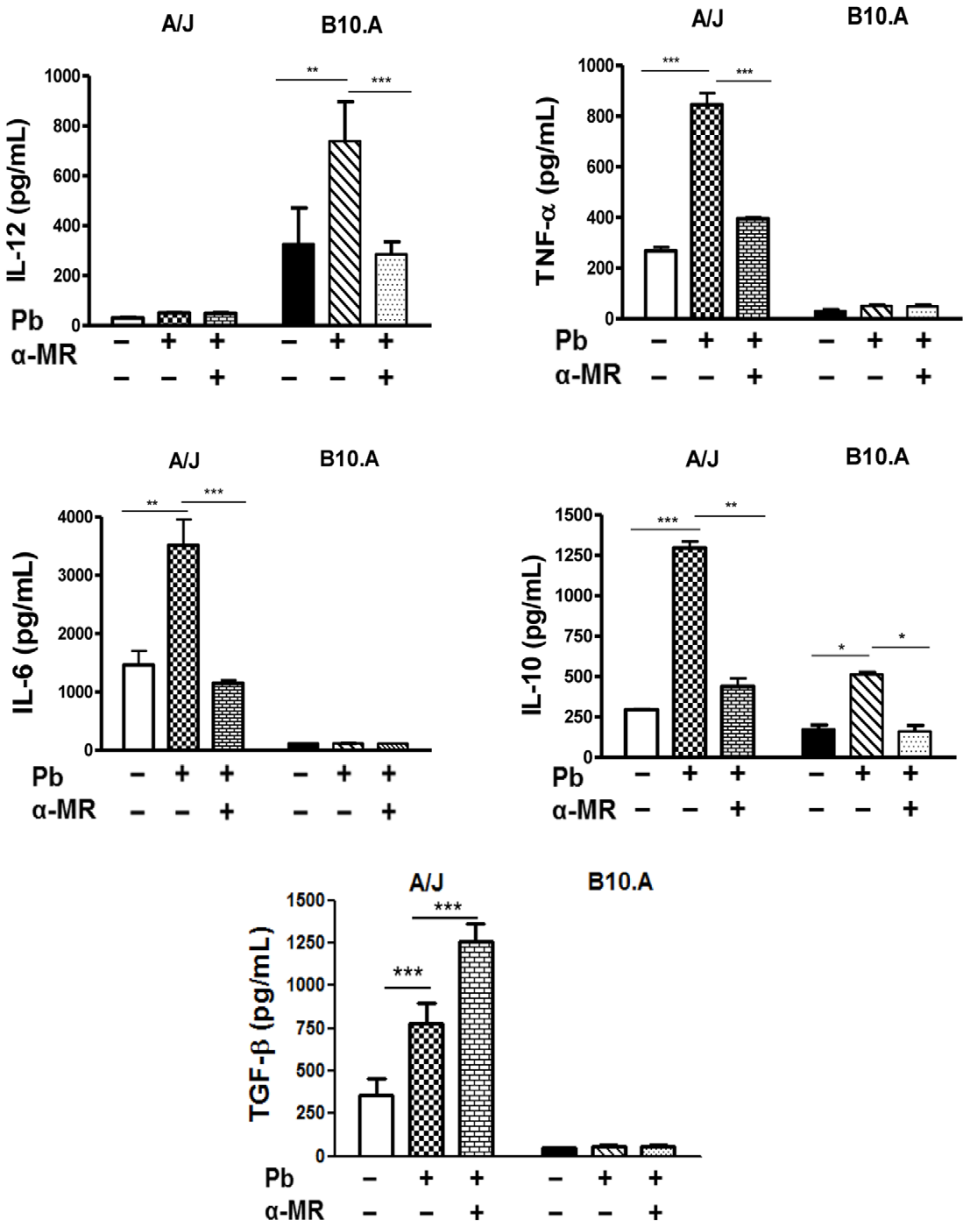
MR signaling controls the different patterns of cytokines produced by A/J and B10.A macrophages. Macrophages from A/J and B10.A mice were treated by anti-MR antibodies (20 µg/mL) for 30 min. After, some cultures were challenged with viable *P. brasiliensis* yeasts (1∶25, fungus:macrophages ratio) for 48 h. Supernatants were removed and used for cytokines measurements by ELISA. Data are means ± SEM of triplicate samples from two experiment determinations. (*P<0.05, **P<0.01 and ***P<0.001).

The influence of MR blockade on the production of cytokines was also assessed. Culture supernatants obtained from fungicidal assays using anti-MR antibodies and IFN-γ-primed macrophages were tested for the presence of IL-12, IL-6, TNF-α, IL-10 and TGF-β. As depicted in Figure 6, blockade of the MRs abolished IL-12 production by B10.A macrophages, whereas the absence of TNF-α and IL-6 were observed in the supernatants from A/J macrophages. Anti-MR antibodies also abolished IL-10 production by both B10.A and A/J macrophages. In contrast, higher levels of TGF-β were detected in the supernatants from MR-blocked A/J macrophages. Therefore, the MR appears to regulate the typical cytokines produced by A/J and B10.A macrophages, with the exception of TGF-β, which was negatively regulated by MR signaling.

### Blockade of TLR4 and CR3 Reduces the Recovery of *P. brasiliensis* in Phagocytic and Killing Assays but does not Alter NO Production by B10.A and A/J Macrophages

**Figure 7:**
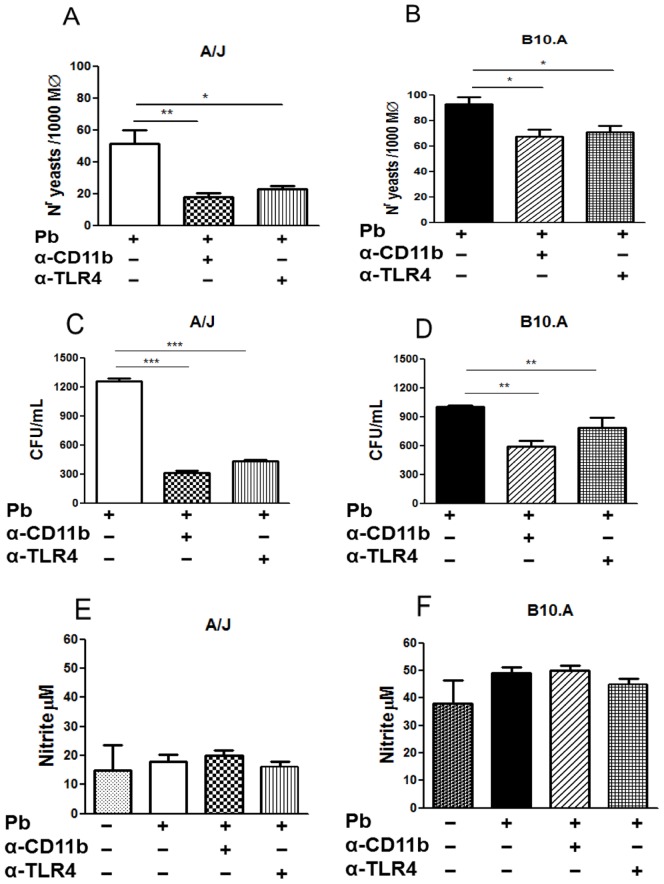
TLR4 and CR3 control the recognition of *P.brasiliensis* yeasts by A/J than B10.A macrophages. Macrophages from A/J and B10.A mice were untreated or treated with anti-TLR4 and anti-CD11b antibodies (10 µg/ml) for 30 min. and then infected or not with *P. brasiliensis* yeasts cells. (A, B) Adherence/ingestion activity, (C, D) fungicidal activity, and, (E, F) NO production were assessed as described before. Data are means ± SEM of triplicate samples from two experiment determinations. (*P<0.05, **P<0.01 and ***P<0.001).

Macrophages from A/J and B10.A mice were treated with anti-TLR4 and anti-CD11b antibodies (10 µg/ml), and their phagocytic, fungicidal and NO production abilities were assessed. Both antibodies reduced the phagocytosis/adherence of yeasts to macrophages (Figure 7A, B). Reduced CFU counts were also observed in anti-CD11b antibody and anti-TLR4 antibody treated cultures, suggesting an increase in fungicidal ability (Figure 7C, D). However, neither of the treatments altered NO production by A/J and B10.A macrophages, indicating that the low adherence exerted a major effect in the fungal recovery in killing assays (Figure 7E, F). Similar results were found with IFN-γ primed macrophages (data not shown).

### TLR4 and CR3 Blockade by Anti-TLR4 and anti-CD11b Antibodies Modulate the Production of Cytokines by A/J and B10.A Macrophages

**Figure 8:**
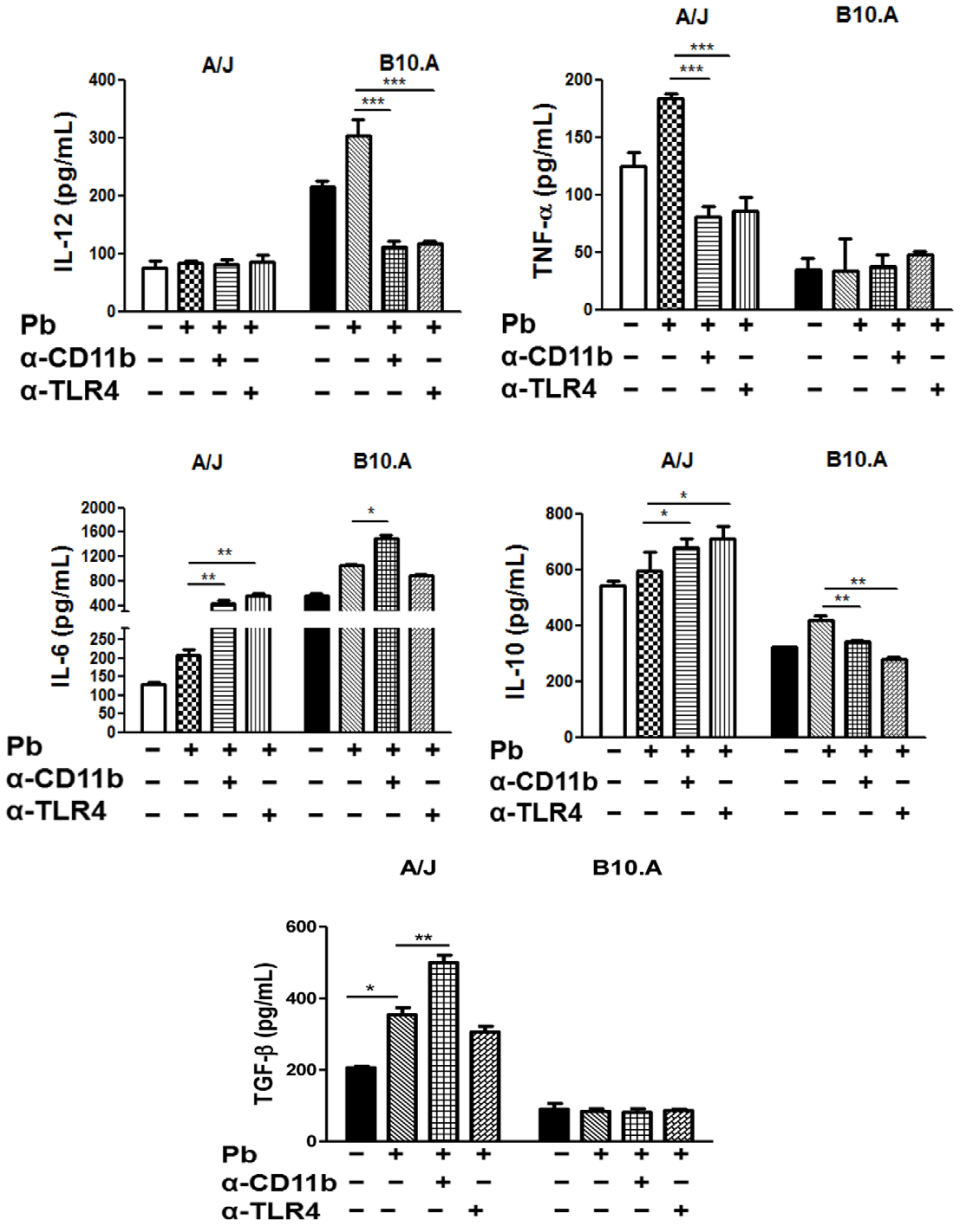
TLR4 and CR3 blockade by anti-TLR4 and anti-CD11b antibodies modulated the cytokines production of *P. brasiliensis* infected A/J and B10.A macrophages. Macrophages from A/J and B10.A mice were untreated or treated with anti-TLR4 and anti-CD11b antibodies (10 µg/ml) for 30 min., infected or not with *P. brasiliensis* yeasts cells, and then cultivated for 48 h. The levels of cytokines were assessed by ELISA in the cell supernatants. Data are means ± SEM of triplicate samples from two experiment determinations. (*P<0.05, **P<0.01 and ***P<0.001).

To further characterize the effect of TLR4 and CR3 blockade by anti-TLR4 and anti-CD11b antibodies, supernatants from the killing assays were tested for the presence of cytokines. As depicted in Figure 8, the blockade of TLR4 and CR3 by specific antibodies abolished both IL-12 and IL-10 synthesis by *P. brasiliensis* infected B10.A macrophages. However, anti-CR3 antibodies significantly increased IL-6 production by these cells. In A/J macrophages, TLR4 and CR3 blockade abolished TNF-α production but increased the levels of IL-6 and IL-10. Furthermore, augmented levels of TGF-β were detected when A/J macrophages were treated with anti-CR3 antibodies.

### Mannan Treatment and *P. brasiliensis* Infection Induce a High Expression of NOS2 in B10.A Macrophages Whereas a High Expression of ARG-1, FIZZI1and YM1 in A/J Macrophages

**Figure 9:**
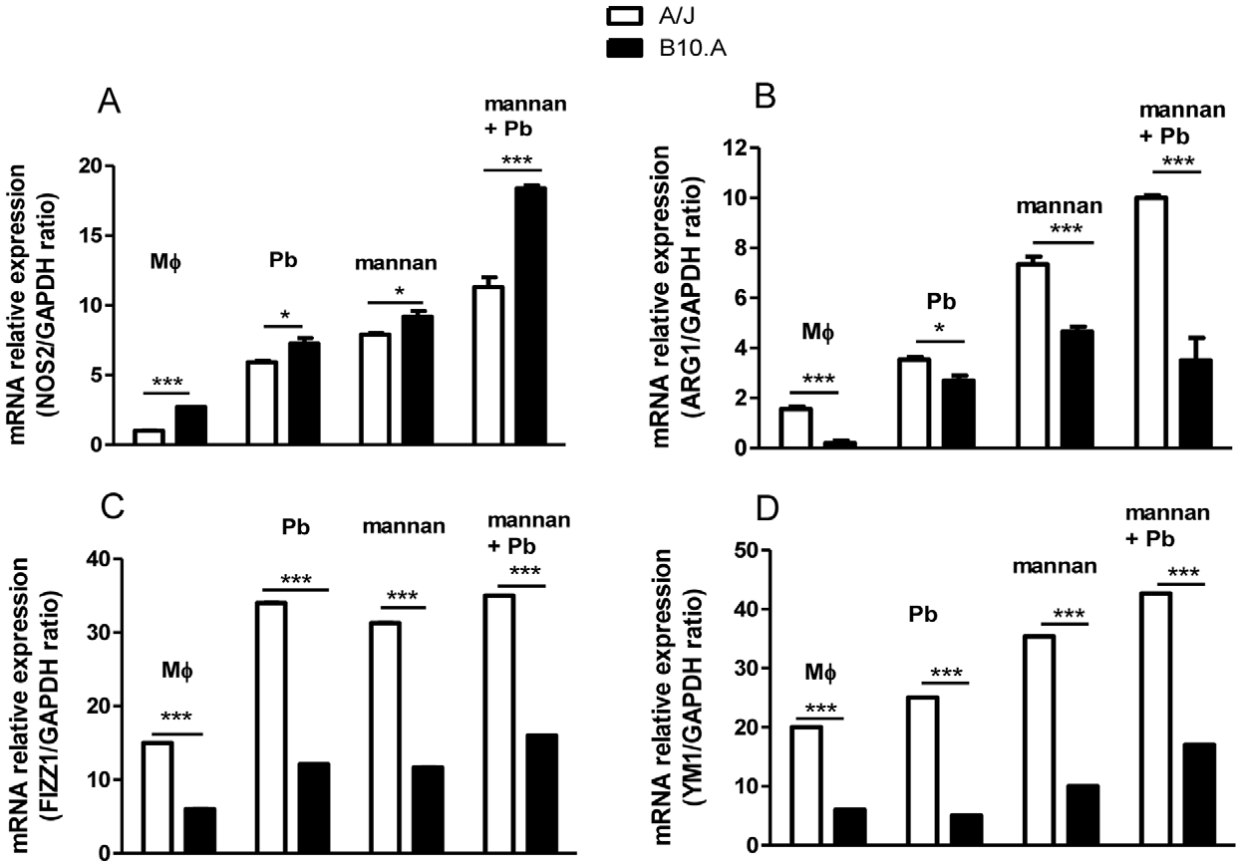
*P. brasiliensis-* and mannan-activated macrophages from susceptible mice preferentially upregulate NOS2, whereas macrophages from resistant mice upregulate ARG1, FIZZ1 and YM1 expression. Quantitative PCR analysis of (A) NO-synthase 2 (NOS2), (B) arginase 1 (ARG1), (C) found in inflammatory zone protein (FIZZ1) and (D) chitinase-like lectin (YM1) mRNA expression. Macrophages from A/J and B10.A mice were untreated or treated by mannan (2.5 mg/mL) for 30 min and cultivated for 12 h. Some cultures were only infected by viable *P. brasiliensis* yeasts (1∶25, fungus:macrophages ratio) for 12 h. Total RNA from macrophages cultures was obtained, reverse transcribed, and cDNA amplified. Real-time PCR was performed using TaqMan universal master mix. Amplified products were normalized to the amount of GAPDH products from in vitro cultivated macrophages. Data represent the means ± SEM of at least 5 mice/group and are representative of two independent experiments. (*P<0.05 and ***P<0.001).

The diverse behavior and pattern of cytokines produced by B10.A and A/J macrophages led us to suppose that mannan and *P. brasiliensis* induced a prevalent pro-inflammatory or M1-like differentiation in B10.A macrophages but a predominant anti-inflammatory of M2-like differentiation in A/J macrophages. M1 macrophages are associated with NO production and enhanced microbicidal activity, whereas M2 macrophages promote healing and tissue repair but show impaired microbicidal activity [45]. Because M1 macrophages are associated with elevated NO-synthase 2, whereas M2 macrophages with arginase-1, FIZZI1 and YM1 [45]–[49] production, we assessed the mRNA expression of these M2 gene markers by B10.A and A/J macrophages following mannan treatment and *P. brasiliensis* infection. As shown in Figure 9A, macrophages from uninfected and infected B10.A mice expressed higher levels of NOS2 after mannan treatment in comparison with A/J macrophages. In contrast, compared with B10.A macrophages, cells from A/J mice expressed much higher levels of arginase-1, FIZZ1 and YM1 after mannan and *P. brasiliensis* treatments (Figure 9B–D).

**Figure 10:**
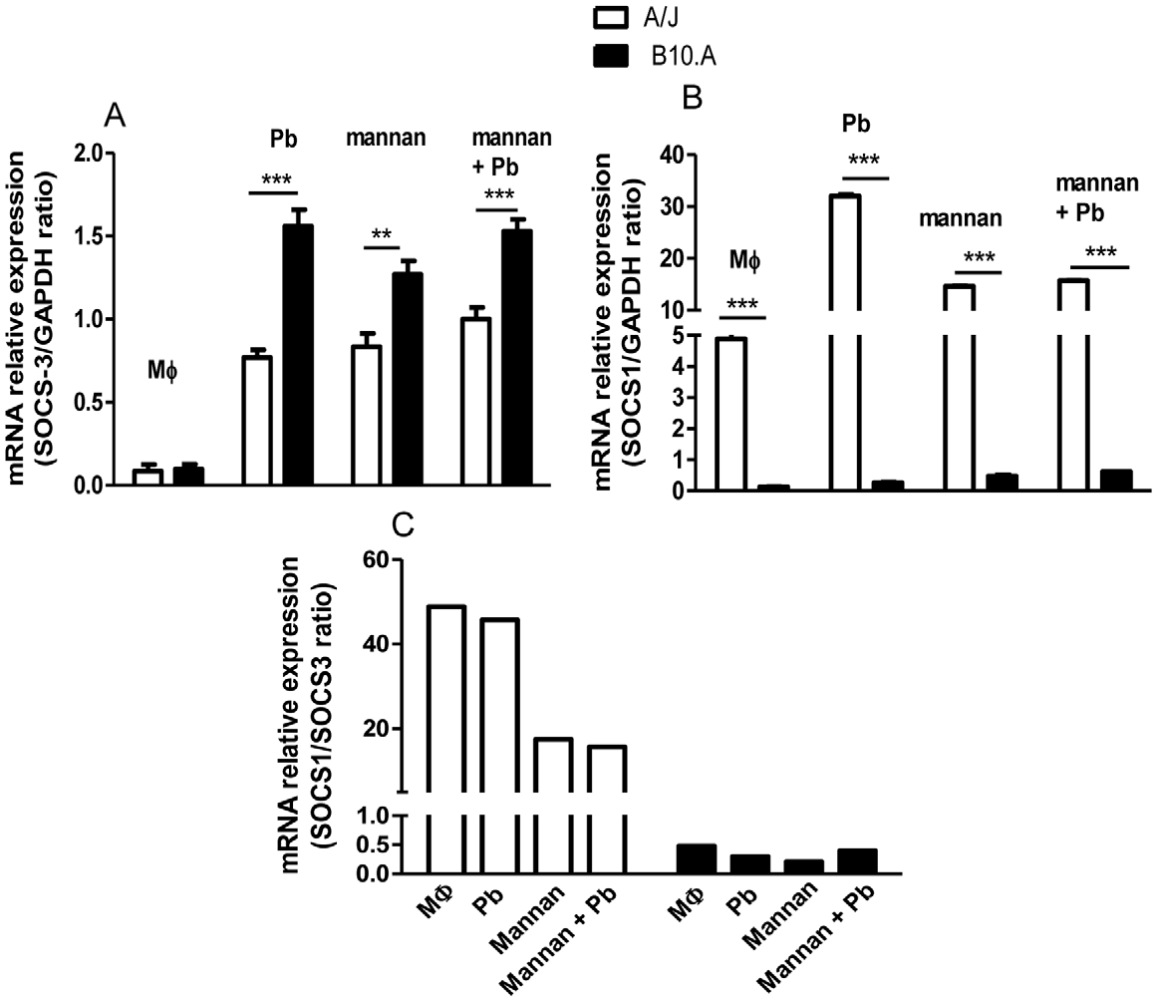
*P. brasiliensis-* and mannan-activated macrophages from susceptible mice preferentially upregulate SOCS3 whereas macrophages from resistant mice upregulate SOCS1 expression. Quantitative PCR analysis of (A) suppressor of cytokine signaling-3 (SOCS3), (B) SOCS1 mRNA expression. Graph (C) represents the SOCS1/SOCS3 ratio. Macrophages from A/J and B10.A mice were untreated or treated by mannan (2.5 mg/mL) for 30 min and cultivated for 12 h. Some cultures were only infected by viable *P. brasiliensis* yeasts (1∶25, fungus:macrophages ratio) for 12 h. Total RNA from macrophages cultures was obtained, reverse transcribed, and cDNA amplified. Real-time PCR was performed using TaqMan universal master mix. Amplified products were normalized to the amount of GAPDH products from in vitro cultivated macrophages. Data represent the means ± SEM of at least 5 mice/group and are representative of two independent experiments. (**P<0.01 and ***P<0.001).

The expression of SOCS1 and SOCS3, which do not define M1/M2 polarization [50] but are involved in the iNOS: arginase balance in both M1 and M2 macrophages, were used to better determine the phenotypes of A/J and B10.A macrophages. As depicted in Figure 10A, mannan and *P. brasiliensis* induced higher levels of SOCS3 in B10.A than in A/J macrophages. In contrast, untreated and treated A/J cells express elevated levels of SOCS1 which was almost not produced by B10.A macrophages (Fig. 10B). Because SOCS1 is specifically involved in the induction of arginase by M2 macrophages, a high ratio SOCS1/SOCS3 can be used to identify M2 macrophages which express high levels of arginase-1. In contrast, a low SOCS1/SOCS3 ratio can be exploited to identify M1 macrophages which fail in the SOCS1 induction but present high levels of SOCS3 expression and a prevalent iNOS induction [46], [51] As shown in Figure 10C, A/J cells showed a high SOCS1/SOCS3 ratio suggesting a M2 differentiation. In contrast, in B10.A macrophages this ratio was lower than 1.0, indicating a prevalent M1 differentiation. Interestingly, our data also demonstrated that untreated A/J macrophages already show an increased expression of arginase-1 and SOCS1 indicating a natural or innate M2 polarization. Although not so evident, B10.A macrophages show an increased NOS2 expression suggesting a tendency to the M1 phenotype.

## Discussion

The mannosyl residues present in several pathogen membranes or cell walls are sensed by multiple PRRs, including MR, TLR2, TLR4, dectin-2, langerin and DC-SIGN [1], [2], [10]–[15]. The MR recognizes mannose and fucose on the surface of pathogens, is highly expressed in alternatively activated macrophages and mediates the phagocytosis of pathogenic organisms. Cytoplasmatic motifs have not been described for the MR on macrophages, but its activation can result in the production of pro-or anti-inflammatory responses, depending on the ligand and phagocyte [13], [15], [48]. The MR was previously shown to interact with a wide range of microorganisms and proteins, including *P. brasiliensis* and gp43, a glycoprotein secreted by yeast cells [1], [2], [10], [25], [31], [33], [49]. Here, we clarified the role of MR, CR3 and TLR4 in the phagocytic and fungicidal mechanisms of macrophages from resistant and susceptible mice to *P. brasiliensis* infection. Our main conclusion is that the MR functions as a phagocytic receptor that promotes the adherence, ingestion and fungicidal ability of A/J and B10.A macrophages. CR3 and TLR4 appear to be mainly involved in the adherence of *P. brasiliensis* cells to macrophages and in the modulation of cytokine production. Importantly, when activated by mannan or *P. brasiliensis* yeasts, B10.A and A/J macrophages produced particular patterns of cytokines, indicating the existence of complex mechanisms of fungal recognition and cell signaling that are likely induced by the concerted action of multiple PRRs.

In this study, mannan was shown to activate macrophages of resistant and susceptible mice and to increase their fungicidal and secretory abilities. Using two different assays, diminished ingestion/adherence of fungal cells to macrophages was observed. Additionally, the concomitant decrease of CFU counts and augmented NO production in the killing assay suggest that, among the mannosyl recognizing receptors studied, the MR exerts adherence and endocytic roles and is intensely involved in the characteristic patterns of cytokines produced by both the B10.A and A/J macrophages. Interestingly, mannan treatment exerted a more prominent effect on macrophage activation than *P. brasiliensis* yeasts; however, both stimuli led to a similar cytokine response. The B10.A cells secreted IL-12 and a small amount of IL-10, whereas A/J cells produced TNF-α, IL-6, IL-10 and TGF-β, suggesting that the MR acts synergistically with other PRRs. Importantly, anti-MR antibodies abolished all cytokine production by both mouse strains, except for TGF-β production by A/J cells, indicating a major contribution of this receptor to the diverse patterns of cytokines produced. TLR2 may be the other mannosyl recognizing receptor that is engaged by A/J macrophages, as evidenced by the significant reduction of its expression after ligand binding. Interestingly, TLR2 signaling was also shown to be involved in the enhanced synthesis of IL-10 by *P. brasiliensis* infected macrophages [28], and it may have contributed to the increased IL-10 secretion observed with A/J macrophages. In contrast, CR3 was concomitantly mobilized with TLR2 in B10.A macrophages, indicating that the MR plays a prevalent role in A/J cells whereas CR3 in B10.A macrophages. The endocytic role of the MR and the major adherence function of the CR3 help to explain the different patterns of macrophage activation reported here. In a study of human macrophages, the endocytosis of the PRRs TLR2, TLR4 and dectin-1 after *P. brasiliensis* infection was described [32]. This indicates that the membrane complexes are internalized after fungal binding.

*P. brasiliensis* yeasts are potent activators of the complement system and use the alternative, classical and lectin pathways to opsonize fungal cells with iC3b fragments and produce chemotactic factors [26], [52]. In this study, opsonization of yeast cells by complement components was avoided by the use of heat inactivated fetal calf serum. It is well known that the iC3b fragment of the complement system is the main ligand for CR3, and LPS from Gram negative bacteria is the main ligand for TLR4 [9], [32], [33]. However, CR3 and TLR4 were also shown to interact with the mannose residues of carbohydrate components of fungal cells [13], [49]. The specific blockade of CR3 and TLR4 decreased the adherence of *P. brasiliensis* cells to A/J and B10.A macrophages, but did not interfere with NO production. This suggests that the lower numbers of CFU recovered from the killing assays were due to the decreased adherence of the fungal cells on the macrophages. Indeed, CR3 and TLR4 are known as adherence, but not phagocytic receptors [12], [53]. The latter function is only observed when these receptors act synergistically with additional stimuli [54], such as activation by TNF-α or GM-CSF [55], [56]. In the A/J mice, the MR, which was seen in reduced levels after macrophage infection by *P. brasiliensis*, appears to be primarily involved in the endocytic activity of these cells. In the B10.A macrophages, the MR appears to play a less important role than it does in the A/J cells; however, it may also influence fungal endocytosis in collaboration with CR3 and TLR4. Our work did not exclude the participation of other PRRs in the recognition of *P. brasiliensis*, and they may have also contributed to the macrophage response. Although diminished expression of CR3 was detected only after *P. brasiliensis* infection of B10.A cells, anti-CD11b antibody treatment of the A/J and B10.A macrophages led to complete abrogation of TNF-α and IL-12 production, indicating that this receptor cooperates with the MR in determining the different patterns of cytokines that are produced by these cells. In murine blastomycosis, macrophages were shown to use CR3 to recognize and ingest the BAD1 antigen and inhibit the TNF-α production required for host immunoprotection [57]. Therefore, is tempting to hypothesize that the lack of TNF-α production by B10.A macrophages may be associated with the prevalent mobilization of CR3 by B10.A macrophages upon *P. brasiliensis* infection and mannan treatment.

The absence of IL-12 and TNF-α secretion was also observed after TLR4 blockade, suggesting that these cytokines are controlled by multiple receptors in a redundant way. However, the contribution of each receptor to macrophage activation appears to be different, as indicated by the particular cytokine responses. Anti-CR3 and anti-TLR4 antibodies induced increased synthesis of IL-6 and IL-10, but blockade of CR3 and MR increased the synthesis of TGF-β. These data indicate that each mannosyl receptor has a different contribution to macrophage activation that appears to depend on their initial expression on the surface of macrophages and their interaction with *P. brasiliensis* ligands. Despite the down modulation promoted by the CR3 and MR in the secretion of TGF-β by A/J macrophages, the alveolar macrophages from this mouse strain are primarily regulated by this cytokine, which defines their poor fungicidal ability and impaired NO synthesis [22].

The innate sensing of the *P. brasiliensis* cell wall by macrophage PRRs helps to determine the host immune response and outcome of infection [28]. Mannan-containing components and alpha-1,3-glucan occupy the outermost layer of the *P. brasiliensis* cell wall [34]–[37]. The proposed structure for the cell wall of *P. brasiliensis* supports the important role of mannosyl recognizing receptors described here. These receptors may interact with the external layer of *P. brasiliensis* yeasts that are rich in mannan and GPI-linked mannoproteins. However, our data clearly showed that soluble mannan induced a stronger activation of macrophages than *P. brasiliensis* infection, suggesting poor accessibility of the mannan-containing components in the yeast cells. Interestingly, Rappleye et al. [58] described the inhibitory activity of the external alpha-1,3-glucan layer of *Histoplasma capsulatum* in the recognition of the fungal beta-1,3-glucan by dectin-1 receptors of macrophages, suggesting that alpha-1,3 glucan, which is a common structure of primary fungal pathogens, may function as an escape mechanism for fungi [58], [59]. Therefore, it is possible that the outer alpha-1,3-glucan layer of *P. brasiliensis* also acts to impair fungal recognition by the MRs.

The interaction of mannan and *P. brasiliensis* with B10.A and A/J macrophages appears to be regulated downstream of ligand binding by divergent cell signaling pathways. This would explain the different cytokines profiles detected in this study. To our knowledge, the cell signaling pathway induced by the MR is not known, but it is associated with the production of pro- and anti-inflammatory cytokines [11], [60]. Our data confirm our previous studies demonstrating the prevalent secretion of IL-12 by B10.A macrophages [23], [24]. Interestingly, *P. brasiliensis* infection induced the internalization of TLR4 on B10.A macrophages, which is in agreement with our previous studies demonstrating that TLR4 and MyD88 signaling were responsible for IL-12 synthesis upon *P. brasiliensis* infection of C57Bl/6 macrophages [28], [29]. On the other hand, the prevalent secretion of IL-6 and TGF-β by A/J macrophages suggests a possible role for mannosyl recognizing receptors in the induction of Th17 immunity and regulatory T cells, which were previously shown to be regulated by these cytokines [27], [60]. Because Th17 immunity has been associated with protective immunity in paracoccidioidomycosis [27] and other fungal infections [25], [60], it is possible that the macrophage activation by mannosyl recognizing receptors could contribute to the immunoprotection of A/J mice.

Our previous studies [22]–[24], [61] and data presented here suggest that *P. brasiliensis* infection and mannan treatment induce in B10.A macrophages a M1-like polarization whereas in A/J macrophages a M2-like differentiation. Indeed, M1 or classically activated macrophages are defined by their high production of NO and microbicidal ability against most microorganisms. They produce high levels of pro-inflammatory cytokines (particularly IL-12) and inhibit TGF-β synthesis and IL-6 signaling impairing Th2 and Th17 differentiation [45]. On the other hand, M2 or alternatively activated macrophages are defined by their poor microbicidal activity, prevalent production of anti-inflammatory cytokines, as well as high expression of MR, arginase-1, FIZZ1 and YM1 [47]. The profile of M2 macrophages is associated with clearance of helminthic infections and the resolution of inflammatory processes [45], [62].

Although B10.A macrophages did not develop all the features of M1 differentiation, the IL-12 secretion, the efficient fungicidal activity, the elevated levels of NO, the low expression of arginase-1 and SOCS1 mRNA, as well as the strong expression of iNOS and SOCS3 mRNA led us to classify these cells as M1-like macrophages. M1-like appears to be appropriate because, apart from the elevated secretion of IL-12, the synthesis of almost all of the other pro-inflammatory cytokines analyzed was suppressed. The nearly total absence of TNF-α and IL-6 in the B10.A supernatants might be attributed to the inhibitory activity of excessive or sustained NO production. Indeed, NO may inhibit the expression of numerous cytokines by macrophages and other inflammatory cells. These include cytokines critical for the development of the inflammatory process and activation of the immune response, such as IL-1β, TNF-α, and IL-6 [63]. The inhibitory effect is exerted by the S-nitrosylation of transcription factors, including NFkB/IkB and JAK/STAT [64]. In agreement, our previous studies demonstrated that *P. brasiliensis*-infected A/J macrophages secrete high levels of TNF-α and produce low NO concentrations, whereas the opposite was observed with B10.A macrophages. Notably, iNOS inhibition by L-NAME restored the TNF-α production by B10.A cells [24].

A/J macrophages, in contrast, develop an “M2-like” behavior when challenged with *P. brasiliensis* and mannan. They have a poor control of *P. brasiliensis* growth, secrete low levels of NO, produce high levels of inhibitory cytokines (TGF- β and IL-10), express elevated levels of arginase-1 mRNA, and show a high ratio of SOCS1/SOCS3 mRNA. All these features are linked to the M2 profile of macrophages [65]–[67]. However, the A/J macrophages also secrete TNF-α, which is usually associated with the pro-inflammatory behavior of phagocytes, and IL-6, which exerts either pro- or anti-inflammatory effects depending on the experimental setting. We can suppose that the secretion of pro-inflammatory cytokines by A/J cells was influenced, at least partially, by the reduced NO secretion that did not impair macrophage activation following the concomitant interaction of several PRRs with multiple cell wall components of *P. brasiliensis*. Alternatively, the differentiation of two opposing subsets of macrophages would explain the behavior of A/J cells: a pro-inflammatory subset mainly involved in the synthesis of TNF-α and another anti-inflammatory subset mainly associated with TGF-β secretion. If confirmed by further experiments, this possibility would recapitulate the behavior of A/J dendritic cells where tolerogenic plasmacytoid and pro-inflammatory myeloid phenotypes were induced by *P. brasiliensis* infection (Pina et al, submitted).

As a whole, our data indicate that B10.A and A/J macrophages do not develop all typical markers of M1 and M2 polarized cells defined by IFN-γ/LPS or IL-4/IL-3 activation, respectively [68]. The differentiation of A/J macrophages adheres much more to the M2b and M2c profiles described for M2 macrophages induced by PRRs ligands, immune complexes or IL-10 activation [69]. In accordance, our data were obtained with mannan- and *P. brasiliensis-*activated A/J and B10.A macrophages which express different levels of mannosyl recognizing receptors which appear to be differently engaged in fungal recognition. MR, a typical M2 marker [66]–[67], seems to be preferentially involved in the “M2 like” polarization of A/J cells, whereas CR3 appears to be preferentially used by B10.A macrophages.

This apparently paradoxical M1-like activation of macrophages associated with susceptibility and M2-like with resistance to *P. brasiliensis* infection was also seen with alveolar macrophages [23] and dendritic cells (Pina et al. submitted). However, during in vivo *P. brasiliensis* infection, the poor fungal clearance observed in A/J mice was followed by a late development of delayed type response, a typical Th1 immunity [22], [61]. In addition, our recent studies have also shown an up-regulated Th17 immunity tightly controlled by suppressive Treg cells in A/J mice [38]. These findings are consistent with the elevated MR, SOCS1, TGF-β, TNF-α and IL-6 production by A/J macrophages and DCs. Our data are also consistent with the early efficient control of fungal burdens of B10.A mice [22] which, however, evolve to suppressed CD4^+^ T cell immunity due to the excessive NO production [24].

In conclusion, macrophages from mice that are resistant to *P. brasiliensis* and those from susceptible mice appear to use different combinations of several mannosyl-recognizing receptors resulting in different profiles of cell activation that likely exert profound influences on the innate and adaptive immunities developed by these mouse strains. However, the *P. brasiliensis* ligands for these mannosyl recognizing receptors are yet to be fully determined.
